# Population Size Estimation of Men Who Have Sex With Men in Rwanda: Three-Source Capture-Recapture Method

**DOI:** 10.2196/43114

**Published:** 2023-03-27

**Authors:** Elysee Tuyishime, Catherine Kayitesi, Gentille Musengimana, Samuel Malamba, Hailegiorgis Moges, Ida Kankindi, Horacio Ruisenor Escudero, Ignace Habimana Kabano, Tom Oluoch, Eric Remera, Angela Chukwu

**Affiliations:** 1 African Center of Excellence in Data Science University of Rwanda Kigali Rwanda; 2 Division of Global HIV and Tuberculosis Center for Global Health US Centers for Disease Control and Prevention Kigali Rwanda; 3 HIV/AIDS, Sexually Transmitted Infections and Viral Hepatitis Division Rwanda Biomedical Center Kigali Rwanda; 4 Key Population Surveillance Team, Epidemiology and Surveillance Branch Division of Global HIV and Tuberculosis, Center of Global Health US Centers for Disease Control and Prevention Atlanta, GA United States

**Keywords:** population size, men who have sex with men, nationwide, capture-recapture, RDS, three-source, Rwanda, HIV

## Abstract

**Background:**

Globally, men who have sex with men (MSM) continue to bear a disproportionately high burden of HIV infection. Rwanda experiences a mixed HIV epidemic, which is generalized in the adult population, with aspects of a concentrated epidemic among certain key populations at higher risk of HIV infection, including MSM. Limited data exist to estimate the population size of MSM at a national scale; hence, an important piece is missing in determining the denominators to use in estimates for policy makers, program managers, and planners to effectively monitor HIV epidemic control.

**Objective:**

The aims of this study were to provide the first national population size estimate (PSE) and geographic distribution of MSM in Rwanda.

**Methods:**

Between October and December 2021, a three-source capture-recapture method was used to estimate the MSM population size in Rwanda. Unique objects were distributed to MSM through their networks (first capture), who were then tagged according to MSM-friendly service provision (second capture), and a respondent-driven sampling survey was used as the third capture. Capture histories were aggregated in a 2k–1 contingency table, where k indicates the number of capture occasions and “1” and “0” indicate captured and not captured, respectively. Statistical analysis was performed in R (version 4.0.5) and the Bayesian nonparametric latent-class capture-recapture package was used to produce the final PSE with 95% credibility sets (CS).

**Results:**

We sampled 2465, 1314, and 2211 MSM in capture one, two, and three, respectively. There were 721 recaptures between captures one and two, 415 recaptures between captures two and three, and 422 recaptures between captures one and three. There were 210 MSM captured in all three captures. The total estimated population size of MSM above 18 years old in Rwanda was 18,100 (95% CS 11,300-29,700), corresponding to 0.70% (95% CI 0.4%-1.1%) of total adult males. Most MSM reside in the city of Kigali (7842, 95% CS 4587-13,153), followed by the Western province (2469, 95% CS 1994-3518), Northern province (2375, 95% CS 842-4239), Eastern province (2287, 95% CS 1927-3014), and Southern province (2109, 95% CS 1681-3418).

**Conclusions:**

Our study provides, for the first time, a PSE of MSM aged 18 years or older in Rwanda. MSM are concentrated in the city of Kigali and are almost evenly distributed across the other 4 provinces. The national proportion estimate bounds of MSM out of the total adult males includes the World Health Organization’s minimum recommended proportion (at least 1.0%) based on 2012 census population projections for 2021. These results will inform denominators to be used for estimating service coverage and fill existing information gaps to enable policy makers and planners to monitor the HIV epidemic among MSM nationally. There is an opportunity for conducting small-area MSM PSEs for subnational-level HIV treatment and prevention interventions.

## Introduction

Globally, men who have sex with men (MSM) continue to bear a disproportionately high burden of HIV infection [1]. In sub-Saharan Africa, same-sex behaviors have become an area of interest for HIV research. The results from recent studies indicate the widespread existence of MSM groups across sub-Saharan Africa and high rates of HIV infection among this population [2]. As countries approach HIV epidemic control in sub-Saharan Africa, attention has shifted to key affected populations, including female sex workers (FSW), people who inject drugs (PWID), transgender women, and MSM who are at a higher risk of HIV acquisition [3].

These key populations account for less than 5% of the global population, but they and their sexual partners comprised 70% of new HIV infections in 2021 [4]. MSM living in sub-Saharan African countries, where homosexual activity has often been severely criminalized, are hard to reach with HIV prevention programs. Currently, the risk of getting an HIV infection is nearly five times higher among MSM in countries where there is criminalization for homosexuality compared to that for MSM in countries where no criminalization for homosexuality occurs [5].

Biologically, unprotected receptive anal sex poses a much higher risk than unprotected receptive vaginal sex, whether the receptive partner is male or female; the risk of HIV transmission during anal intercourse may be approximately 18 times greater than that during vaginal intercourse [1]. By this fact, MSM are at an extremely high risk of becoming infected with HIV. The global estimated HIV prevalence among MSM is 21%, with 6% in sub-Saharan countries, ranging from 3.8% up to 31% [6,7]. In addition, people with marginalized sexual or gender identities or behaviors sometimes lack the ability to protect themselves from HIV infection due to structural factors, including self-stigmatization, discrimination, and lack of access to information and services [8]. The prevalence ratios are particularly elevated in West and Central Africa as well as in low-prevalence countries [9].

Rwanda is in the East African region, bordering four countries: Tanzania, Uganda, the Democratic Republic of the Congo, and Burundi. The country is divided into five administrative regions, including four provinces, the city of Kigali, and 30 districts as another subnational unit level. Rwanda experiences a mixed HIV epidemic, which is generalized in the adult population, with an adult (15-49 years) HIV prevalence stabilized at around 2.6%, and aspects of a concentrated epidemic among specific key populations at higher risk of HIV infection, with a rate of 45% among FSWs [10] and 6.5% among MSM [11]. In Rwanda, MSM are considered one of the key populations for HIV prevention and care in the HIV and AIDS national strategic plan [12].

Although the national strategic plan suggests a special emphasis on key populations and that the epidemic is likely concentrated among these populations, limited data exist to estimate the actual population size at the national scale [12]. To date, no nationwide study has been performed to estimate the number of MSM in Rwanda. However, the Project San Francisco study performed in 2018 using a service multiplier and unique object identifier approach estimated the size of MSM in the city of Kigali and surrounding areas to be 8411, ranging between 6760 and 11,151 [11]. Lack of reliable estimates of the MSM national population size misses important information to determine the denominators to estimate need, coverage, and the spread of new infections that policy makers and planners use to monitor HIV epidemic control.

In response to this information gap, we performed a national population size estimation of MSM in Rwanda from October to December 2021, aimed at providing national-level and provincial-level estimates.

## Methods

### Study Population

The study population included adult males aged 18 years and older who self-reported as gay/bisexual or having had anal sex with a male in the last 12 months and lived primarily in Rwanda during the past 12 months. By living primarily in Rwanda, we refer to having spent most of their time in Rwanda over the last 12 months, regardless of any possibility of traveling out of the country. The study excluded any MSM who were not willing to voluntarily participate.

### Study Design and Setting

Estimating the size of hard-to-reach populations has long been a challenging task due to the fact that sampling frames often do not exist for these populations, some populations are dynamic both in time and space, and members may be afraid to identify themselves in the community. There are multiple methods suggested to estimate the size of populations without a sampling frame, including venue-based sampling [13], time-location sampling [14], respondent-driven sampling (RDS) [15], multiplier methods [16], the network scale-up method [17], successive sampling-population size estimation [18], and capture-recapture (CRC) [19]. No gold-standard method currently exists, and many methods have been used, each with different strengths and weaknesses.

The CRC method has been shown to be useful in estimating the size of hard-to-reach populations [20-22]. Based on its statistical foundation, CRC, as an empirical population size estimate (PSE) method, produces PSEs with higher precision. Research suggests that, compared with two-source CRC, conducting CRC with multiple (three or more) sampling/capture rounds strengthens the design, produces more robust estimates, and relaxes the CRC requirement (assumption) of sample independence [23]. The CRC method is increasingly being used by several researchers in epidemiology and public health [24-26], and this method was previously used to estimate the national size estimate of FSWs in Rwanda, demonstrating that acquired implementation experiences are crucial for such estimates [27].

We used the three-source capture-recapture (3S-CRC) method to estimate the size of MSM in Rwanda. CRC consists of marking a random portion of the population of interest at the first encounter. Subsequently, another portion of the population is drawn to observe how many were marked initially. The greater the number of those tagged, the smaller the PSE. Tagging can be replicated multiple times as desired. The 3S-CRC approach has been shown to be a robust method in estimating a population without a sampling frame, such as FSWs, MSM, and PWID [28], and is described elsewhere in more detail [29]. The MSM 3S-CRC began with a capture stage. During this stage, members of the MSM population were “encountered” and then “marked” by providing a memorable and easily identifiable gift with a specific design that is hard to find on the local market. After a 1-week interval, a second capture (recapture) was initiated by offering MSM-friendly services to MSM countrywide. During the third capture (recapture), an RDS approach was used [30,31] with specific questions incorporated to identify those MSM encountered during the previous capture occasions.

During capture one, MSM key informants were selected by MSM community-based organizations (CBOs) to facilitate the distribution of unique objects within MSM associations and groups and those who are neither members of any association nor MSM group. A list of MSM associations and groups was developed with their corresponding numbers of members in all provinces of the country: 14 from Eastern province, 12 from the city of Kigali, 10 from Southern province, and 8 from each of the Northern and Western provinces. Each province was assigned a different unique color of the unique objects to be distributed. The number of objects to be distributed within each association or group was determined by the probability proportional to the association or group size; within an association or a group, systematic sampling was found to be appropriate to determine who receives the unique object according to the existing list of MSM association or group members. The approach comprises determining a sampling interval as well as a random start on the list of MSM association or group members.

Each selected MSM member within an association or a group was given three unique objects: one for himself and another two to hand out to other MSM he knows but who do not belong to any association or group. The distribution process for unique objects within an MSM association or group was facilitated by a well-trained team of distributors with MSM key informants’ support. Branded keychains valued at no more than US $3 were used as unique objects. MSM were provided with the study objectives and advised to keep the received unique objects in a safe place as they may be asked to show them later for verification. This activity took 1 week to be completed.

There were possibilities that some objects could not be successfully distributed and not returned as well as multiple objects given to the same person. To minimize any related bias on the PSEs, a debrief was given to the MSM key informant as well as the MSM association/group members focusing on study objectives; eligibility criteria to receive the object; object distribution process; and key considerations, including prompting the object receiver to check if he had not been approached by someone else in the same study context to avoid duplication. In addition, the monetary value of the unique object was set to US $3 to minimize the intention for the object distributor to keep the objects for themselves or the object receiver being willing to receive more than one object. The object distribution process was monitored daily by the object distributor and MSM key informant to report the number of objects distributed successfully as well as those not successfully distributed, which were returned physically.

The following week, capture two was initiated, where MSM were tagged using selected specific MSM-friendly services. The selection process for services was facilitated by MSM key informants and their CBOs. Given that MSM are given health services through usual health facilities but equipped with MSM-friendly settings and packages, the latter were used to offer specific MSM-friendly services, including distribution of condoms and lubricants. For this purpose, 23 health facilities were selected across the country where an MSM key informant and a health worker who usually serve MSM at the same facility were assigned to provide the selected service to MSM and to record corresponding information in the study purposes. The key informant role was to serve as the receptionist and to facilitate screening to create a welcoming and friendly environment for MSM, while the health worker served to provide services and record necessary information. Prior to the second capture, community mobilizers served on the mobilizing of upcoming service provision to MSM to increase awareness and to break the fear and stigma of participation. Those who accepted the offered services were counted as captured and were asked whether they had received the distributed unique object during the previous week. Responses (yes/no) were recorded depending on whether the MSM had the gift with him or was able to correctly identify the unique object received.

Capture two was followed by the Integrated Behavioral and Biological Surveillance Survey (IBBSS) using RDS [32], which served as the opportunity for the third capture. RDS is a variant of chain-referral sampling that employs Markov-chain theory and the theory of biased networks to reduce biases generally associated with chain-referral methods [31]. It was proven that even though sampling begins with a purposively chosen group of initial subjects, as is the case for most chain-referral samples, the composition of the ultimate sample is wholly independent of those initial subjects [33]. During capture three, all MSM recruited by their peers using the RDS approach were counted as captured. There were eight study sites distributed according to the administrative provinces: one in each of the Northern, Southern, and Eastern provinces; two in the Western province; and three in the city of Kigali. MSM who met the study eligibility criteria and were well-connected and respected among their MSM peers were selected as seeds and marked the beginning of each referral chain. During this study, the investigator contacted the seed via the implementing partners, including nongovernmental organizations and CBOs working with the MSM population. Three seeds were selected at each study site for a total of 24 seeds.

During the RDS survey, participants were asked whether they had received unique objects during capture one and/or had received provided services during capture two during the second week of study implementation. Several prompts were used to confirm prior participation, including having received the object physically or being able to correctly identify the received object on a laminated card with images of multiple objects. MSM were not allowed to participate more than once in the same round; an interoperable fingerprint system was used to verify that each participant was recorded only once during capture three and to detect any duplication within the same site and across all study sites.

Fingerprint machines were installed at each study site and connected to the internet for easy and real-time data synchronization. Once a fingerprint was recorded, it was automatically converted into alpha-numerical codes and transferred to the central server to synchronize all study site–level data. With this installed system, we could identify any MSM trying to reregister his fingerprint at the same site or at a different site. The fingerprint machines converted the recorded fingerprints into alpha-numerical codes with no reverse backway possibility, and then the alpha-numerical codes were stored as participant identifiers to ensure participants’ confidentiality.

### Sample Size and Sampling

For the first two initial captures, the sample size was based on Rwanda Population-Based HIV Impact Assessment 2019 [34], where the proportion of all males aged 18+ years with at least one male sexual partner in the last 12 months by province (0.30% for the city of Kigali, and 0.16%, 0.08%, 0.24%, and 0.24% for the Eastern, Northern, Southern, and Western provinces, respectively) was obtained to estimate the pooled and provincial-level stratified sample size. Assuming a design effect of 1.5, a precision of 0.5%, and adjusting for a 15% loss of coupons estimated from a previous CRC-related study [27], we calculated the required minimum sample size. We estimated a total of 2705 objects needed to be distributed across provinces as follows: 803 in the city of Kigali, 586 in Western province, 658 in Southern province, 219 in Northern province, and 439 in Eastern province during each of the first two captures.

For the RDS survey, the sample size calculation was based on the results from the previous IBBSS conducted in Rwanda in 2020. The formula for the sample size calculation for an RDS study was used according to the method of Salganik [35], assuming an MSM HIV prevalence of 11.3% in the city of Kigali, 6.4% in Western province, 1.4% in Southern province, 3.1% in Northern province, and 1.2% in Eastern province; a design effect of 1.5; precision *w* 0.025; and a nonresponse rate of 10%. We estimated the required minimum total sample size of 2210 across the country distributed by province as follows: 1027 in the city of Kigali, 613 in Western province, 141 in Southern province, 308 in Northern province, and 121 in Eastern province.

All of the estimated sample sizes for each capture and the statistical power were validated using MS-CRC Power Analysis of the shinyrecap application [36].

### Data Management

Data were captured using 3 electronic forms (capture one, capture two, and capture three forms) using tablets with data-entry forms designed using Open Data Kit (ODK) [37]. The tablets were password-protected to ensure that only the study team had access to the data. Other data were captured using Microsoft Excel sheets, which were also password-protected. Data cleaning, entailing deduplication, review of data consistency, and completion of any missing data, was performed daily.

Unique identifiers were used to ensure that study participants were only enrolled once. Once data were deduplicated, all identifiers were removed prior to creating the final data sets for analysis.

### Statistical Analysis

In preparation for analysis, participant-level data from ODK were exported into R v.4.0.5 for Windows, and cleaning steps were performed based on preset exclusion criteria and logical flow. Data were analyzed by province. Aggregated data sets detailing counts of each capture/recapture combination were produced for each subset. The Bayesian nonparametric latent-class model, which is flexible and able to accommodate various forms of heterogeneity in capture probabilities, was used to produce the final PSE with 95% credible sets (CS) from aggregate data sets.

All analyses were performed using the latent-class model for capture-recapture (LCMCR) package in R v.4.0.5 for Windows [38,39]. LCMCR is a novel Bayesian nonparametric method for estimating the size of hard-to-reach populations from CRC data. This method is based on a Dirichlet process mixture, capable of accommodating complex patterns of heterogeneity of captures, and can transparently modulate its complexity without a separate model selection step [40,41]. The Bayesian nonparametric latent-class approach posits that the population is divided into several groups with members in each group having the same homogeneous capture probability. The number of homogeneous strata in a population is uncertain and covariates that identify those classes may not be available. Thus, the strata are said to be latent and strata identities are treated as missing data. Estimation is naturally accomplished using mixtures of distributions [42].

Uninformative priors (ie, those with minimal influence on the inference and dominated by the likelihood function) were specified for the Dirichlet process parameters α and γ (0.25, 0.25). We used K=5 latent classes; 10,000 samples from the posterior distribution were drawn with a burn-in of 10,000 iterations and a thinning interval of 1000 iterations to specify the Markov Chain Monte Carlo (MCMC) sampling. Convergence of the MCMC sampling was assessed using trace plots and a histogram of the posterior probability distribution for population size.

Median PSEs with 95% CS for 3S-CRC were produced overall and by province. To facilitate the interpretation of results and application of estimates for programs, the highest density intervals (HD Interval package in R) are presented. Analysis outputs include the median PSEs with 95% CS.

### Ethical Considerations

The survey received ethical approval from the local Institutional Review Board, Rwanda National Ethics Committee. It was also reviewed in accordance with the US Centers for Disease Control and Prevention (CDC) human research protection procedures and was determined to be research with CDC nonengaged. For the PSE study component, we received a waiver of the informed consent requirement for all MSM participants because no personally identifiable information was collected; however, verbal consent was sought since the data collected were restricted to only recording whether the MSM accepted the gift during subsequent capture.

Since same-sex relationships remain stigmatized in Rwanda, we anticipated a small risk of physical and/or verbal violence in cases where a study participant was identified in the community. Therefore, the study investigator ensured that the study was conducted anonymously to protect the identity of participants and ensure confidentiality of the data collected; no names or any other personally identifiable information were recorded anywhere. Completed questionnaires (identifying individuals by only identification numbers) were kept with the study coordinator during the fieldwork. All forms containing any study information were kept in a locked cabinet accessible by only authorized study personnel. Additionally, the electronic data set identified by the unique code was password-protected and accessed by authorized personnel using a computer backed up to a server located at Rwanda Biomedical Center. Furthermore, for all study members participating in human subjects research and data collection, it was a requirement for the study team to have participated in trainings on human subjects research, confidentiality, and interviewing techniques before commencing study activities. A confidentiality agreement was signed by all study investigators, coinvestigators, and data collectors.

## Results

### Capture One

**Figure 1:**
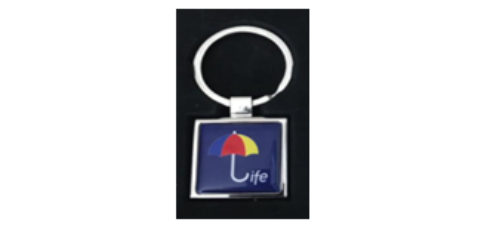
Example of a unique object (keychain) distributed in capture one.

Keychains with unique designs were distributed to the MSM through their corresponding associations, groups, and key informants (Figure 1). Table 1 summarizes the results from capture one; a total of 2465 out of the 2723 objects (90.53%) were successfully distributed.

**Table 1:** Provincial-level object distribution breakdown.

Location	Objects assigned, n	Objects successfully distributed, n	Objects not distributed successfully and returned, n
Eastern province	636	558	78
City of Kigali	894	885	9
Northern province	166	150	16
Southern province	585	515	70
Western province	442	357	85
Total	2723	2465	258

### Capture Two

MSM-friendly services were provided at 23 health facilities that typically have a key population service package countrywide. Lubricants and condoms were distributed during the capture-two period, during which a total of 1340 of the anticipated 2705 MSM (49.54%) came for health services at health facilities. Out of the 1340 MSM who came for the services, 1314 (98.06%) met the inclusion criteria and were offered the services, and among them, 721 (54.87%) were identified as having received the distributed unique object during the previous week. Table 2 provides the province-level breakdown for capture two.

**Table 2:** Provincial-level service provision among men who have sex with men (MSM) in capture two.

Location	Health facilities, n	Anticipated MSM to be offered services, n	MSM received at health facility, n	MSM received offered services, n	MSM received distributed unique object during the previous week, n
Eastern province	5	439	343	337	211
City of Kigali	5	803	510	497	185
Northern province	1	219	28	25	4
Southern province	7	658	291	291	195
Western province	5	586	168	164	126
Total	23	2705	1340	1314	721

### Capture Three

During capture three, RDS was used for participant recruitment. Every MSM recruited during RDS was screened for eligibility and considered as captured during capture three once he consented to participate. A total of 2211 MSM were captured during this capture occasion. Among those captured during capture three, 422 (19.09%) were identified as having received distributed unique objects during capture one, whereas 415 (18.77%) were identified as having received MSM-friendly services during capture two. Table 3 provides the provincial-level breakdown for capture three.

**Table 3:** Provincial-level data among men who have sex with men (MSM) in capture three.

Location	Anticipated MSM to be captured, n	MSM captured, n	MSM received distributed unique object, n	MSM received provided MSM-friendly services, n	MSM received both unique object and MSM-friendly services, n
Eastern province	121	126	50	64	36
City of Kigali	1027	1,021	128	124	42
Northern province	308	303	20	18	4
Southern province	141	152	42	54	28
Western province	613	609	182	155	100
Total	2210	2211	422	415	210

### Population Size Estimation

**Figure 2:**
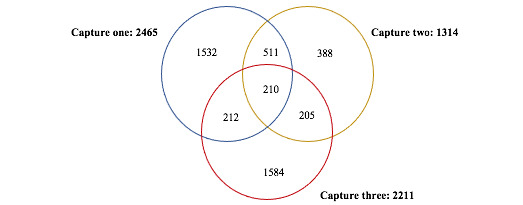
Venn diagram representing individual capture results and overlaps between capture occasions. Numbers represent the numbers of men who have sex with men captured at each round.

Overall, we sampled 2465, 1314, and 2211 MSM in capture one, two, and three, respectively. There were 721 recaptures between captures one and two, 415 recaptures between captures two and three, and 422 recaptures between captures one and three. There were 210 MSM captured in all three captures. The Venn diagram in Figure 2 provides the summary results for all three capture occasions and overlaps.

Before conducting the CRC analysis, we explored dependency between captures by testing for homophily in the RDS recruitment chain based on the capture history variable, finding a homophily value of 1.016596, which indicates nondependency.

**Figure 3:**
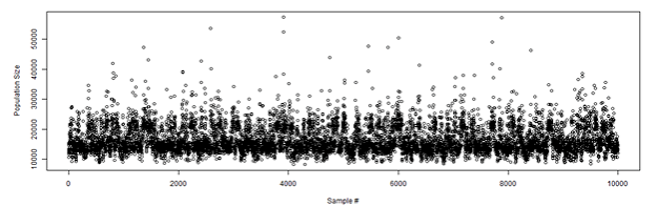
Trace plot for the population size estimate.

**Figure 4:**
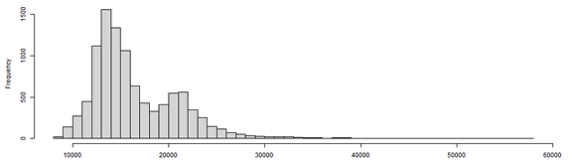
Histogram of the posterior probability distribution for population size.

The trace plot in Figure 3 presents the simulation results over 10,000 samples, demonstrating the population size distribution for a converging simulation result based on the random noise shape observed between the values 10,000 and 20,000 on the Y-axis. Figure 4 presents the posterior distribution (ie, population size distribution) provided by the model.

The final MSM PSEs for the overall and provincial levels are presented in Table 4. The median from the posterior probability distribution is used as the point estimate and 95% CS are used to describe uncertainty.

We estimated the overall population of MSM in Rwanda to be 18,100 (95% CS 11,300-29,700), with the majority living in the city of Kigali (7842, 95% CS 4587-13,153). The MSM PSEs were similar for the remaining 4 provinces (Northern, Southern, Eastern, and Western provinces).

**Table 4:** Population size estimate (PSE) of men who have sex with men, Rwanda 2022.

Location	Proportion estimate^a^, % (95% CI)	Median PSE (95% CS^b^)
Eastern province	0.3 (0.3-0.5)	2287 (1927-3014)
City of Kigali	2.7 (1.6-4.6)	7842 (4587-13,153)
Northern province	0.5 (0.2-1.0)	2375 (842-4239)
Southern province	0.4 (0.3-0.6)	2109 (1681-3418)
Western province	0.3 (0.3-0.5)	2469 (1994-3518)
Overall	0.7 (0.4-1.1)	18,100 (11,300-29,700)

^a^Proportion estimates of men were based on the 2012 Census data by National Institute of Statistics of Rwanda 2021 population size projections.

^b^CS: credibility set.

## Discussion

This MSM PSE presents the first use of the 3S-CRC approach to estimate MSM population size on a nationwide scale in Rwanda. The 3S-CRC method provided an estimate for MSM in the city of Kigali of 7842 (95% CS 4587-13,153), which was similar to the previous estimate obtained by “Project San Francisco” of 8411 (95% CS 6760- 11,151) [11]. The slight differences in the 2018 and 2021 Kigali MSM PSEs can be explained by considering the geographical coverage and difference in methodologies used. The distribution of MSM in each of the remaining 4 provinces was fairly uniform and lower than the estimates in Kigali. Differences in estimates of the MSM population size distribution across the country may also reflect long-term movement patterns among MSM, from rural to urban as well as from smaller to larger urbanized contexts [43].

To some degree, MSM size estimates are influenced by the proportion of MSM who may have decided to not participate in the study due to potential privacy concerns, a potentially significant element given the burden of stigma and heteronormative behavioral expectations (eg, marriage and parenting) for MSM in Rwanda. Our overall MSM PSE represents 0.7% (95% CI 0.4%-1.1%) of the total adult male population in Rwanda based on 2012 Census data collected by National Institute of Statistics of Rwanda 2021 population size projections. The 2020 World Health Organization (WHO) and Joint United Nations Program on HIV/AIDS (UNAIDS) technical brief recommends the revision of the MSM PSE for those countries, using an MSM PSE less than 1% of total adult males based on the region [28]. The UNAIDS monitoring system through 2019 estimated a global median proportion of adult men who had sex with another man in the previous year of 1.9% across 38 low- or middle-income countries, and this proportion was estimated at 1.45% in Eastern and Southern Africa, where Rwanda is located [44,45]. Accordingly, the estimate from the current study aligns with the WHO recommendation regarding MSM PSEs [7].

Different approaches are being used for size estimation of hidden populations, each with various strengths and limitations, and there is currently no consensus on a gold-standard method [46]. The traditional CRC method employs two captures; however, extra captures can be added to increase the number of data points from which estimates are created, resulting in more stable and robust PSEs [47]. The 3S-CRC method has been commonly used in epidemiology to estimate the size of key populations targeted by health interventions for certain health conditions owing to its mathematically grounded and defensible results [23,48,49]. Several studies have used the 3S-CRC method to estimate the size of certain population groups without a sampling frame, including FSW, MSM, and PWID [28,50-52]. The final estimate for this study was based on the 3S-CRC data set. In summary, four major assumptions must be met for CRC to give reliable population estimates: individual captures are independent, the population is closed, the capture history is correct for all members of the target population, and the chance of getting caught is homogeneous [53].

To minimize dependencies between captures, we used different distribution settings for each capture occasion. During the first capture, members of the MSM population were tagged by the keychain provided through their corresponding associations, groups, and key informants. In the second capture, MSM were tagged by being offered MSM-friendly services through health facilities that usually serve MSM nationwide. In the third capture, we used the RDS method, in which all recruited MSM were considered as captured. For all three captures, a 1-week time interval was used between two consecutive capture occasions to minimize recall bias and to fulfill the population closeness assumption. At each capture of the first two capture rounds, unique object distribution and MSM-friendly services provision procedures included a random aspect to ensure that the chance of getting caught was homogeneous. However, our estimates might be limited with missing a random sampling aspect during the third capture round where RDS was used.

There are several plausible constraints to the design of our estimation activity. A possible limitation is the underlying 3S-CRC assumptions that might have influenced the validity of our findings, leading to reduced accuracy of population sizes and wide confidence ranges. In particular, we employed unique objects as a tagging strategy to protect the anonymity of sampled populations. However, not all individuals were carrying the received object at the subsequent capture occasion, complicating the identification of recaptures. Furthermore, we had to assume that the person presenting the object is the same person who received the object (an essential limitation present in anonymous sampling–based CRC). We tried to mitigate these limitations by offering the opportunity to identify the correct object from a laminated card with several pictures including the correct unique object for those presenting without unique objects. There was a possibility of guessing or having seen the object and therefore biasing the PSE downward. To overcome possible participation duplicates at enrollment during the third capture, a biometric system using fingerprint identification was installed and employed across all study sites. We also acknowledge any possible selection bias that might have been influenced by the established study inclusion criteria.

The key strength of our study is that it is powered to provide national- and provincial-level PSEs for MSM in Rwanda for the very first time. Sampling considered administrative provinces as strata and targeted 28 (out of 30) districts in Rwanda, which are nationally representative and reflective of the demography. The selected districts included key urban areas with a high likelihood of expanding the catchment to include participation by MSM based in rural areas. Furthermore, during the third capture occasion, RDS was used giving more confidence in reaching MSM with lower social visibility.

The final estimate of the MSM population size in Rwanda is based on a Bayesian approach to account for the complex patterns of heterogeneity between captures and the aggregation of homogeneous strata into latent classes. While other statistical techniques make reasonably strong assumptions about the structure of the joint distribution of capture patterns, the latent-class Bayesian method is a model-averaging strategy that seeks to estimate the joint distribution as directly as feasible from the data [40].

In conclusion, this study provides, for the first time, an estimated population size of MSM aged 18 years and above in Rwanda. The results will allow national programs and implementation partners to invest in HIV services at a level that is commensurate with need, coverage, and new infections. These data enable policy makers and planners to monitor HIV epidemic control nationwide, specifically among the MSM population, and to plan for other health services such as the prevention and treatment of sexually transmitted infections, among others. While these estimates are usable at the national and provincial levels, further work is needed on small-area estimation to align the PSE results with the intended HIV treatment and prevention interventions at subnational levels among MSM. Furthermore, we acknowledge that there are still limitations of estimating some hard-to-reach MSM groups, which is another potential area for further research.

## Abbreviations

3S-CRC: three-source capture-recapture
CBO: community-based organization
CDC: Centers for Disease Control and Prevention
CRC: capture-recapture
CS: credibility set
FSW: female sex worker
IBBSS: Integrated Behavioral and Biological Surveillance Survey
LCMCR: latent-class capture-recapture
MCMC: Markov Chain Monte Carlo
MSM: men who have sex with men
ODK: Open Data Kit
PSE: population size estimate
PWID: people who inject drugs
RDS: respondent-driven sampling
UNAIDS: Joint United Nations Program on HIV/AIDS
WHO: World Health Organization

## Data Availability

Data are owned by Rwanda Biomedical Center (RBC); all data access enquiries should be directed to RBC through the following email address: info@rbc.gov.rw.
